# A Multiplex PCR/LDR Assay for Viral Agents of Diarrhea with the Capacity to Genotype Rotavirus

**DOI:** 10.1038/s41598-018-30301-3

**Published:** 2018-09-04

**Authors:** Aashiq H. Mirza, Sanchita Das, Maneesh R. Pingle, Mark S. Rundell, George Armah, Ben Gyan, Richard L. Hodinka, Davise H. Larone, Eric D. Spitzer, Francis Barany, Linnie M. Golightly

**Affiliations:** 1 Weill Cornell Medicine, New York, NY USA; 2 Noguchi Memorial Institute for Medical Research, University of Ghana, Legon, Ghana; 3 University of South Carolina School of Medicine Greenville, Greenville, South Carolina USA; 4 Stony Brook University Medical Center, Stony Brook, NY USA

## Abstract

Rotavirus and noroviruses are major causes of diarrhea. Variable rotavirus vaccination efficacy in Africa and Asia is multifactorial, including the diversity of circulating strains and viral co-infection. We describe a multiplexed assay that detects and genotypes viruses from stool specimens. It includes a one-step reverse transcriptase PCR reaction, a ligase detection reaction (LDR), then hybridization of fluorescent products to micro-beads. In clinical samples it detects rotavirus, caliciviruses (sapovirus and norovirus), mixed infections, and genotypes or genogroups of rotaviruses and noroviruses, respectively. The assay also has the capacity to detect hepatitis A. The assay was validated on reference isolates and 296 stool specimens from the US and Ghana. The assay was 97% sensitive and 100% specific. The genogroup was concordant in 100% of norovirus, and the genotype in 91% and 89% of rotavirus G- and P-types, respectively. Two rare rotavirus strains, G6P[6] and G6P[8], were detected in stool specimens from Ghana. The high-throughput assay is sensitive, specific, and may be of utility in the epidemiological surveillance for rare and emerging viral strains post-rotavirus vaccine implementation.

## Introduction

Worldwide diarrheal diseases are major causes of illness and death, particularly in young children^1^. Rotavirus (RoV) and caliciviruses (norovirus [NoV] and sapovirus [SaV]) are primary viral agents that cause these infections^2^, with RoV being the most common cause of severe diarrheal disease. To combat rotaviral illnesses, the World Health Organization (WHO) recommends immunization for all children with one of two vaccines: RV1, a human monovalent strain vaccine (Rotarix; GlaxoSmithKline Biologics, Rixenstart, Belgium) or RV5, a pentavalent bovine-human reassortant vaccine (RotaTeq; Merck Vaccines, Whitehouse Station, New Jersey)^3^. The monovalent RoV vaccine was recently included in the childhood immunization program of Ghana and has shown effectiveness against severe rotavirus diarrhea^4^. The vaccines are designed to elicit neutralizing antibodies against two outer capsid proteins (VP4 and VP7) of the segmented double-stranded RNA virus and form the basis of the RoV binary genotype classification system (VP4 [P type] and VP7 [G type]). Although heterotypic protection against rotavirus genotypes not present in these vaccines has been demonstrated, the mechanisms of protection are not well understood. The reason for the variable immune response between populations is also poorly understood, impairing the development of the next generation of vaccines^5^. Therefore, concerns remain regarding the emergence of new genotypes against which immunity may not be conferred^6^. While certain genotypes predominate, reassortment events and interspecies transmission can result in novel genotypes^7^. In low income countries in Africa where the diversity of circulating strains is high, continued surveillance of circulating genotypes is important to monitor for the emergence of new strains that may limit the efficacy of vaccines^8^.

No antivirals and vaccines are presently available to treat calicivirus infections, and in many countries NoV is becoming the leading cause of viral gastroenteritis in young children^9,10^. NoV and SaV constitute genetically diverse small RNA viruses belonging to multiple genogroups. NoV is divided into genogroups GGI to VI and SaV into genogroups GI to GV. NoV genogroups I, II and IV and SaV genogroups I, II, IV, V have been reported to cause serious infections in humans^11^. While GII.4 is the predominant norovirus strain associated with global pandemics, the emergence of new variants is particularly concerning since they are noted to be associated with more severe outbreaks, such as the Sydney 2012 NoV^12^. Determining the genogroup of circulating NoV is important to understand the epidemiology of the disease.

Recently, diagnostic gastrointestinal panels (“GI panels”) have been developed. Based on multiplexed real-time PCR or molecular arrays, the panels are sensitive and specific pathogen identification tools that are useful in patient management^13–16^. The assays are, however, limited in their ability to detect, identify and genotype or genogroup different viral agents simultaneously^17^. Their use in programs aimed at the determination of the prevalence and diversity of viral genotypes or genogroups is therefore restricted. Rotavirus genotypes may be modified by vaccination efforts^18^. In fact, the failure to detect novel variants and genotypes by established molecular assays is well documented^19–21^. Due to the constant accumulation of point mutations and the emergence of novel genotypes, new and updated molecular assays and techniques are required in order to correctly identify new or emerging strains^21–23^. Knowledge of emerging genotypes of RoV and genogroups of caliciviruses could inform public health efforts to find alternate and/or improved means to combat them^24–29^.

We describe a prototype Reverse-Transcription PCR (RT-PCR)/Ligase Detection Reaction (LDR) assay developed to detect and identify major viral etiological agents of childhood diarrhea (RoV, NoV and SaV), and simultaneously provide information regarding genotypes and genogroups. We describe the performance characteristics of the PCR/LDR assay in the detection and identification of RoV, NoV and SaV from clinical specimens. We further report the use of this assay in characterizing RoV genotypes and the 2 most common NoV genogroups from a repository of stool specimens from children with diarrhea in northern Ghana and the eastern US respectively.

## Results

### Optimization of the PCR/LDR assay

The PCR/LDR assay for the detection and genotyping of agents of viral diarrhea was initially optimized and validated on RNA extracted from 24 previously characterized viral culture supernatants and stool specimens (Table 1).

**Table 1:** Rotavirus culture supernates and Calicivirus stool specimens used in PCR/LDR assay validation.

Strain Name/No.	VP7 G genotype	VP4 [P] genotype	Genogroup	Type of sample/Country of origin
Rotavirus				
Wa	G1	P[8]	—	Culture adapted/USA
DS-1	G2	P[4]	—	Culture adapted/USA
ST3	G4	P[6]	—	Culture adapted/UK
US1205	G9	P[6]	—	Culture adapted/USA
P	G3	P[8]	—	Culture adapted/USA
CDC-28	G9	P[8]	—	Human Stool/USA
CDC-75	G1	P[8]	—	Human Stool/USA
CDC-97	G1	P[8]	—	Human Stool/USA
CDC-39	G9	P[6]	—	Human Stool/USA
CDC-53	G3	P[8]	—	Human Stool/USA
Norovirus				
505	—	—	I	Human Stool/USA
510	—	—	I	Human Stool/USA
5220	—	—	II	Human Stool/USA
382	—	—	II.4	Human Stool/USA
2008747532	—	—	I.3b	Human Stool/USA
2008752327	—	—	I.7	Human Stool/USA
2008747520	—	—	II.6	Human Stool/USA
2008747521	—	—	II.4	Human Stool/USA
2008747522	—	—	II.5	Human Stool/USA
Sapovirus				
2008890578	—	—	I and II	Human Stool/USA
2009725961	—	—	I	Human Stool/USA
2008890878	—	—	IV	Human Stool/USA
2007001367	—	—	V	Human Stool/USA

### Performance of the assay for detection of viral agents in stool

The PCR/LDR assay was used to analyze 296 clinical specimens, 210 of which were confirmed to have one or more viral agents using either real-time PCR or sequencing (Table 2). The assay was able to detect the virus in 203 samples (sensitivity of 97%). There were no false positive results (specificity of 100%). Seven samples failed to amplify (2 SaVs, 1 NoV and 4 RoVs) possibly due to lower RNA content in these samples or due to repeated freeze-thawing and transport. Two samples were found to be co-infected with NoV GGII and RoV using PCR/LDR; this was confirmed by sequencing. Pathogen identification by the PCR/LDR assay was concordant in all positive samples. The genogroup of NoV could be determined by PCR/LDR in 49 out of 50 NoV confirmed positive stool specimens (48 of 48 GGII and one of two GGI, 98% overall sensitivity). NoV was not detected by PCR/LDR (failed PCR amplification) in the second NoV GG1 sample.

**Table 2:** Detection of viruses from 296 clinical stool specimens from the US and Ghana.

Identification of samples	Real-time PCR/Sequencing		PCR/LDR*		Additional virus detected by PCR/LDR
	No. Positive	No. Negative	No. Positive	No. Negative	
Rotavirus	148	0	144	4	2^†^
Sapovirus	12	0	10	2	
Norovirus GI	2	0	1	1	
Norovirus GII	48	0	48	0	
Negative	0	86	0	86	

*Sensitivity and specificity of the PCR/LDR assay for detection of viruses in clinical stool specimens were 97% and 100%, respectively. ^†^Norovirus GGII detected in addition to rotavirus by PCR/LDR in Ghanaian samples were confirmed by sequencing.

The PCR/LDR assay was not designed to differentiate SaV genogroups, however, SaV genogroups GGI (n = 3), GGII (n = 1), GGIV (n = 8), were included in the clinical samples tested by the PCR/LDR assay. The 2 SaV clinical samples not detected were both in genogroup GGIV. The assay had a sensitivity of 83.3% for the detection of SaV (Table 2).

The *in-silico* primer design and optimization assays included and validated the detection of the hepatitis A virus, however, further testing on clinical samples was not performed.

### Determination of RoV genotypes in samples from Northern Ghana

Results of the RoV genotype determination in stool samples by the PCR/LDR assay compared to that of the RT-PCR assay are shown in Table 3. A total of 148 RoV-positive stool specimens were analyzed and discrepancies verified by sequencing of the PCR amplicons when sufficient genetic material was available. In 112 specimens assayed, there were concordant single genotypes while in 13 specimens mixed concordant G- and P- genotypes were obtained. Eighteen specimens showed only partial concordance between the typing methods due to the presence of multiple genotypes and/or inability to definitively determine the G- or P- type.

**Table 3:** Comparison of G and P genotypes detected by PCR/LDR and RT-PCR in rotavirus positive stool specimens.

Number of specimens	Genotype determination by			
	PCR/LDR		RT-PCR^22^	
	G-type	P-type	G-type (VP7)	P-type (VP4)
57	G1	P[8]	G1	P[8]
6	G1	P[6]	G1	P[6]
1	G1	P[U]	G1	P[6]
5	G1	P[U]	G1	P[8]
1^A^	G1	P[U]	G1	P[U]
34	G2	P[6]	G2	P[6]
1	G2	P[4]	G2	P[4]
8	G3	P[6]	G3	P[6]
1	G3	P[U]	G3	P[6]
2	G9	P[8]	G9	P[8]
1^B, E^	G6	P[6]	GU	P[6]
1^B,E^	G6	P[U]	GU	P[8]
8	G1+2	P[6]	G1+2	P[6]
1^A^	G1+2	P[4]	G1+2	P[4]
1^A^	G1+2	P[U]	G1+2	P[4]
1	G1+8	P[6]	G1+8	P[6]
1	G1+9	P[8]	G1+9	P[8]
2	G3+9	P[6]	G3+9	P[6]
1	GU	P[U]	G3	P[8]
1	GU	P[U]	G2	P[6]
1^B,C,F^	GU	P[U]	GU	P[U]
1^B^	GU	P[6]	GU	P[6]
1^D^	G1	P[4]	G1+2	P[8]
1	G2	P[4]	G1+2	P[4]
1	G2	P[6]	G1+2	P[6]
1^C^	G1	P[U]	G1	P[U]
4	G1+3	P[6]	G3	P[6]
2	NVA	NVA	G1	P[8], P[6]
1	NVA	NVA	G2	P[6]
1	NVA	NVA	G2+3	P[6]
Total (148)				

^A^P-type confirmed as P[4] by sequencing. ^B^RT-PCR VP7 gene consensus primers produced a PCR product; genotype specific PCR primers did not produce a gene product. ^C^RT-PCR VP4 gene consensus primers produced a PCR product; genotype specific PCR primers did not produce a gene product. ^D^P[8] confirmed by sequencing. ^E^G-type confirmed as G6 by sequencing. ^F^G-type confirmed as G3 by sequencing. NVA indicates no virus amplified by PCR primers. GU and P[U] refers to samples in which virus was detected but either the G- or P-type, respectively, could not be determined. For PCR/LDR testing this is due to lack of adequate signals for genotyping per the algorithm described in Suppl Fig. 1.

Three samples were untypeable for both G- or P-type by PCR/LDR. Two were characterized as G3P[8] and G2P[6], respectively, by conventional RT-PCR. One could not be typed by RT-PCR. Sequencing confirmed the G-type to be G3 but the P-type could not be determined.

Our assay showed a high concordance rate in determining both P- and G-genotypes. RoV G-type determination by PCR/LDR was concordant in the 135/148 samples (91%) as confirmed by RT-PCR or sequencing. Mixed G-types were detected in 18 stool specimens by PCR/LDR, 14 of which were concordant to the results obtained using RT-PCR. Four additional specimens identified as mixed G-types by PCR/LDR were typed as only a single genotype by RT-PCR. In 4 samples RT-PCR detected mixed G-types of which 3 samples were identified as single G-types and in 1 sample no virus was detected by PCR/LDR.

RoV P-type determination by PCR/LDR was concordant in 132/148 (89%) samples as confirmed by RT-PCR or sequencing (Table 3). A mixed P-type was detected by RT-PCR in 2 samples in which PCR/LDR did not detect any virus. In 11 samples, PCR/LDR was unable to confirm the P-type determined by RT-PCR. Both assays had difficulty identifying P[4] isolates. Of the 6 isolates identified as P[4], 1 was untypeable by both methods but the identity was determined by sequencing; 3 were correctly identified as P[4] by PCR/LDR and RT-PCR. In one sample PCR/LDR could not determine the genotype that was determined by RT-PCR and confirmed by sequencing and in another sample PCR/LDR misidentified the genotype as P[4] that was typed correctly by RT-PCR as P[8] and was confirmed by sequencing.

The most common single RoV genotype identified by PCR/LDR was G1P[8] (57/148, 39%) followed by G2P[6](35/148, 24%) (Table 3). Of significant note, we detected RoV G6 in 2 samples: G6P[8] and G6P[6]. RoV G6 has rarely been reported in humans, but is common in cattle^30^. Since the RT-PCR primer mix used for confirmation of genotypes did not include primers for G6, the genotype for these samples was confirmed by sequencing the VP7 amplicon. The partial nucleotide sequences of VP7 regions from both samples have been submitted to GenBank (Accession numbers KP412490 and KP412491).

**Figure 1:**
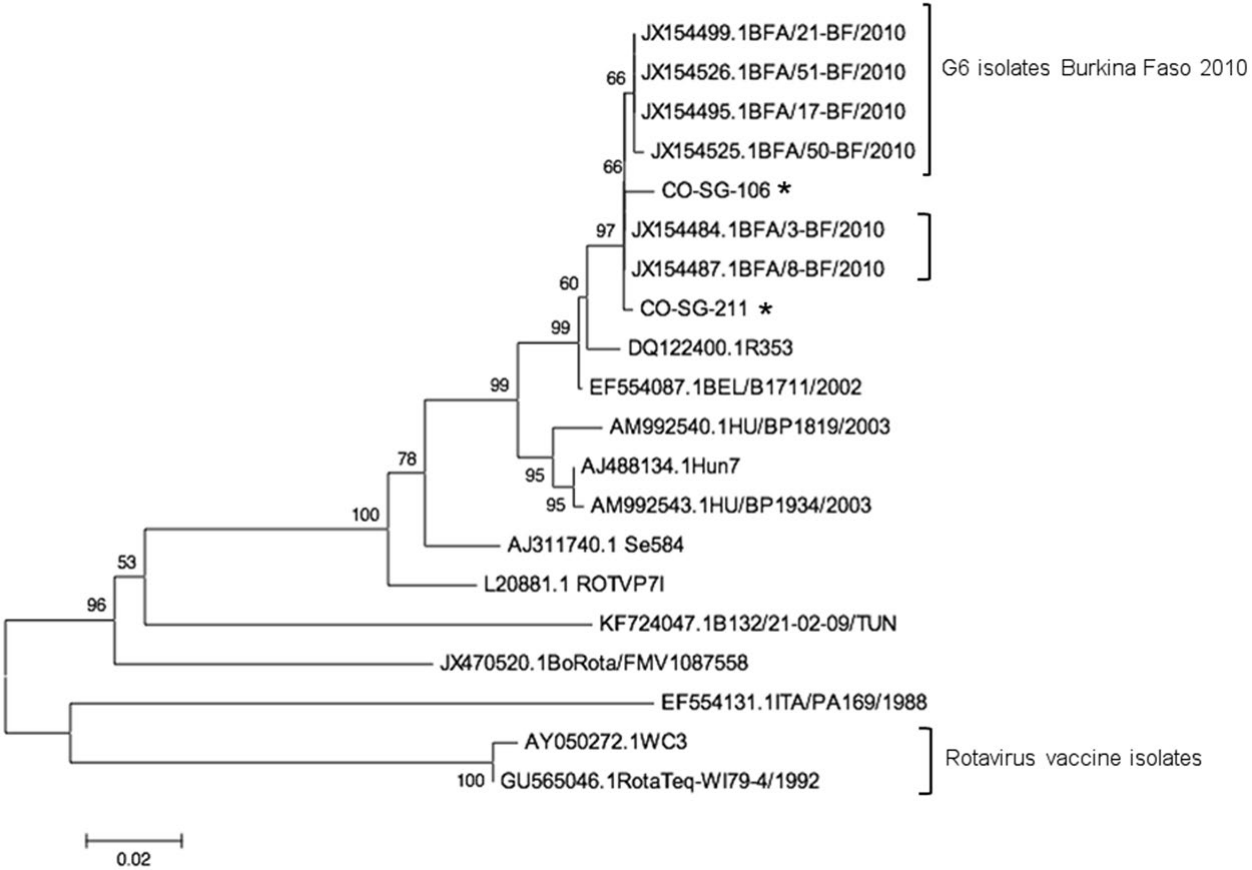
Phylogenetic tree of VP7 gene sequences from G6 rotavirus isolates. *Indicates isolates identified in the current study. Both of these isolates cluster with those recently reported from the geographically proximal country of Burkina Faso in the study of Nordgren *et al*.^30^. The EF554087.1BEL/B1711/2002 isolate is that reported from Mali^33^.

Due to the rarity of this genotype, and to verify that the virus in these samples was not derived from the bovine vaccine strain, a phylogenetic analysis of VP7 nucleotide sequences from the two samples was performed. Comparison to other bovine and human G6 strains available in GenBank revealed a higher degree of homology to human strains (Fig. 1). In addition, they were more closely related to the recent G6P[6] isolates identified among children with diarrhea in Burkina Faso than from that reported earlier from Mali (Fig. 1)^30–33^.

## Discussion

We describe the development and application of a molecular assay for the detection of the most common agents of viral diarrhea in stool. Molecular techniques for the detection of viruses in stool are reliable methods for the laboratory diagnosis of diarrhea. Commercial assays for the multiplex detection of diarrheal pathogens are approved by the US Food and Drug Administration and are used in clinical laboratories^16^. The Luminex xTAG gastrointestinal pathogens panel test based on a multiplex PCR approach has been used to detect RoV in stool samples of children in Ghana^15^. However, such assays are still expensive for routine use in resource-limited areas and multiplexed detection assays have limited genotyping and genogrouping capacity.

The conventional method of genotyping RoV uses semi-nested primers in a 2-step PCR amplification process followed by gel electrophoresis or by sequencing^22,34,35^. The PCR/LDR method employs multiplex reactions and detects RoV, SaV and NoV while simultaneously identifying the genotypes of RoV and the genogroups of NoV in a single test. The assay is performed in a 96-well plate format thus providing information on a large number of specimens in a relatively short period of time. The technology is flexible with the capability of integrating additional primers for the multiplexed identification of new viral variants as they emerge^36^. Additionally, the PCR/LDR technology is capable of high-throughput testing such that population studies and epidemiologic investigations can be performed on a large number of specimens rapidly. The assay is also amenable to automation and has been demonstrated to be adaptable to a microfluidic platform making it an ideal comprehensive system for surveillance studies^37^. These attributes may permit the engineering of an inexpensive and portable device for future iterations of the assay.

Overall, the assay was found to be 97% sensitive and 100% specific for the detection of these viruses from 296 stool specimens from patients with diarrhea collected from 3 centers, one in northern Ghana and two in the US. Stool specimens positive for RoV were obtained from Ghana and the assay was able to detect RoV in 144 of 148 samples. The calicivirus-positive specimens were obtained from two centers in the US and 59 of 62 samples were positively identified. Our assay was designed to identify 2 of the most common genogroups of NoV. The genogroup was determined by PCR/LDR in all specimens in which NoV was detected by the assay (Table 2).

The prototype PCR/LDR assay described was not designed to determine the genogroup of SaV. However, the assay was able to detect the 3 genogroups included in the clinical specimens tested (GG1, GGII, and GGIV). In addition, genogroup V, the least commonly found genogroup in clinical isolates, was not present in the panel of clinical samples tested^38,39^. However, it was detected in a stool sample used to optimize the assay (Table 1). Therefore, the assay was able to detect all clinically relevant SaV genogroups. The slightly lower sensitivity of the assay in the detection of SaV (83.3%) may be due to repeated freeze-thawing and transport of the samples. More extensive testing of clinical SaV samples is indicated but our results are promising.

RoV strains are often non-typeable using standard RT-PCR methods and in some recent studies, especially from Africa. In a review of studies that examined rotavirus isolates from children in Africa as many as 16.3% could not be characterized to G- and P-types^40^. A study by Armah *et al*. investigating the diversity of rotavirus types in West Africa, found a high prevalence of non-typeable strains G-types (11.5%) and P-type (6.8%)^41^. This has been attributed to the possible ongoing genetic re-assortment process among the different genotypes in these regions^41^. Another possibility is the nature of amplification-based assays that seek known genotypes and miss new genotypes arising over time^42^. The PCR/LDR assay was able to correctly ascertain the genotype of RoV in 91% of the strains. The assay is comprehensive in identifying the genotype of the viral pathogen and detecting co-infection with multiple genotypes and viruses in a single assay. Co-infection with more than one virus^14^ has been proposed as a cause of reduced rotavirus vaccine efficacy^18,23^. Infection with mixed genotypes in areas of high endemicity has also been described^40^. Mixed genotypes in Bangladesh have been attributed to high transmission and reassortment^42^. In a study from southern Ghana, both mixed G- (7.3%) and P- (24.2%) genotypes were reported^43^. Therefore, a comprehensive assay such as ours could be of utility in such settings.

*In silico* analysis of our LDR primers indicated that 8 P-types and 10 G-types of RoV would be detected. However, not all combinations are equally distributed globally and therefore not all genetic combinations were included in our analysis of samples from Ghana. Our analysis of specimens from Ghana identified two samples with the RoV G6 type that is rarely seen in humans but is common in animals. Due to the unusual nature of this finding, we sequenced both the VP4 and VP7 genes for confirmation and investigated the phylogeny of the VP7 gene. The unusual combination G6P[6] was first reported in human cases acquired in Mali in 2003 and from France^31,33^. It has been detected only once before in Ghana^32,44^. The G6P[8] combination has been reported from Bulgaria and Bangladesh^45,46^. This is the first report to our knowledge of the G6P[8] type in Ghana; previous cases have been reported from Hungary, Italy, US and Australia^47–50^. The detection of rare and unusual combinations of RoV genotypes from Ghana has been alluded to by Armah *et al*. and proposed to result from a combination of prevalent cultural and epidemiological factors^41^. An unusually high number of G6P[6] isolates has been identified in children with diarrhea in Burkina Faso with some very closely related phylogenetically to isolates from Ghana and Sierra Leone, respectively^30,51^. A recent study by Agbemabiese *et al*.^52^ performed phylogenetic comparison of the genomes of G2P[4] strains isolated from Ghana to the global collection of G2P[4] and African non-G2P[4] isolates that resemble the DS-1 strain. Their study revealed that the dynamic evolution of rotavirus strains through intra-genotypic reassortment events can result in genetic lineages specific to Africa^52^. Our isolate CO-SG-211 (G6P[6]) has >99% identity to the GenBank sequences of isolates from Burkina Faso^30^. Both isolates were also closely related to the other G6 isolates but belonged to a different clade than that of the bovine RoV parent strain used in the formulation of the reassortant vaccine (Fig. 1). Another study in Thailand has also identified human G6 genotype infections^53^. Rahman *et al*. and Nordgen *et al*. suggest that these unusual combinations emerge in the human population due to a recent reassortment event from bovine strains with subsequent adaptation to humans^30,31^.

Current rotavirus vaccines target the prevalent G1-4 and P[8] genotypes. Concerns were initially raised regarding their efficacy given the diverse genotypes of rotavirus^18,23^. However, the vaccines thus far have shown efficacy against strains not contained in the vaccines^54–56^. The epidemiology of circulating rotavirus strains remains of importance in disease surveillance and monitoring, especially following widespread vaccine implementation. Marked change in genetic epidemiology has been reported in Brazil following its mass immunization program and in Gambia in pre-vaccination surveys^57,58^. It has also been proposed that genetic reassortment with the acquisition of human gene segments might result in an increased ability of such recombinant strains to infect and spread among humans^45^. The detection of rare genotypes and co-infection with multiple viruses, as well as the diversity of genotypes, indicates the importance of monitoring the molecular epidemiology of viral diarrhea worldwide. The described assay could be of utility in this effort.

Our study has several limitations. Firstly, the assay was validated on stored specimens that may have undergone repeated freeze-thaw cycles thus compromising the integrity of the RNA present. Secondly, we collected both NoV and RoV stool specimens from a single center in the US and Ghana, respectively. SaV specimens were obtained from a public health laboratory in the midwest region of the US. Thus, the genotype distribution of RoV and genogroup distribution of caliciviruses tested was not diverse. As a consequence, some RoV G- and P-types, such as G5, G10, G12, P[3], P[9], P[10], P[14], were not included in the samples tested. These genotypes are uncommon in Ghana and the US. Their circulation is largely restricted to certain countries in Asia and were thus not present in the specimens tested. Although *in silico* analysis suggests that the assay should detect these genotypes, further studies are needed to validate the primers in clinical samples. Albeit in a small number of samples, the assay was able to detect all the clinically relevant SaV genogroups either in our validation assay or clinical samples. Thirdly, we designed primers to genotype RoV, but the prototype assay was not designed to characterize calicivirus strains to the same extent, although it has the capacity to do so. The assay was designed intentionally in this manner given the importance of RoV genotyping in surveillance post the introduction of rotavirus vaccines. Although, the distribution of NoV and SaV genogroups are important in disease pathogenesis the epidemiology has yet to be described in detail, and in the absence of vaccines, the importance of their relative distribution in the population has not been completely elucidated^59,60^. However, the PCR/LDR technology has the potential to genogroup caliciviruses and further studies can be performed to elucidate the distribution of NoV and SaV in different populations as required.

## Methods

### Ethics Statement

This study was approved by the Institutional Review Boards of Weill Cornell Medicine and the Noguchi Memorial Institute for Medical Research (NMIMR), University of Ghana. All research was performed in accordance with relevant guidelines and regulations.

### Genetically characterized virus strains and stool specimens

Cell culture-adapted human reference rotavirus strains with known G and P types and positive stool specimens containing genetically characterized RoV, NoV and SaV were obtained from the Gastroenteritis and Respiratory Viruses Lab Branch, Centers for Disease Control and Prevention, Atlanta, GA and the United States Department of Agriculture, Agricultural Research Service, Delaware State University.

### Clinical stool specimens

A total of 296 diarrheic stool samples made up of (1) 148 positive for RoV and 86 negative stools obtained from NMIMR, (2) 50 NoV positive stools from Children’s Hospital of Philadelphia and (3) 12 stools confirmed positive for SaV obtained from the Minnesota Department of Health, were used for the assays.

### RNA Extraction

Viral RNA was extracted from 200 µl cell culture supernates or from 10% fecal suspensions in phosphate buffered saline; the fecal suspensions were clarified by centrifugation at 3000 rpm for 10 min prior to RNA extraction using the QIAamp Viral RNA kit (Qiagen, Inc., Valencia, CA), following manufacturer’s instructions with the following modification: 96-well plates from the QIAamp 96 DNA Blood Kits were used in place of individual QIAamp Mini Spin Columns.

### Primer and Assay Design

PCR and LDR primers (Supplemental Tables S1A and S1B) were designed using Oligo 6.0™ software (Molecular Biology Insights, Cascade, CO) and were based on sequences obtained from the GenBank database^36,61,62^. Regions for PCR amplification were within the VP7 and VP4 genes for RoV, the ORF1-ORF2 junction for NoV, and the VP1 capsid region for SaV. The PCR primers designed for detection of the viral agents were tested in uniplex reactions (using groups of primers specific for each viral species) and in multiplex reactions (using all primers in a single reaction). Products of amplification were analyzed on 2% agarose gels.

**Figure 2:**
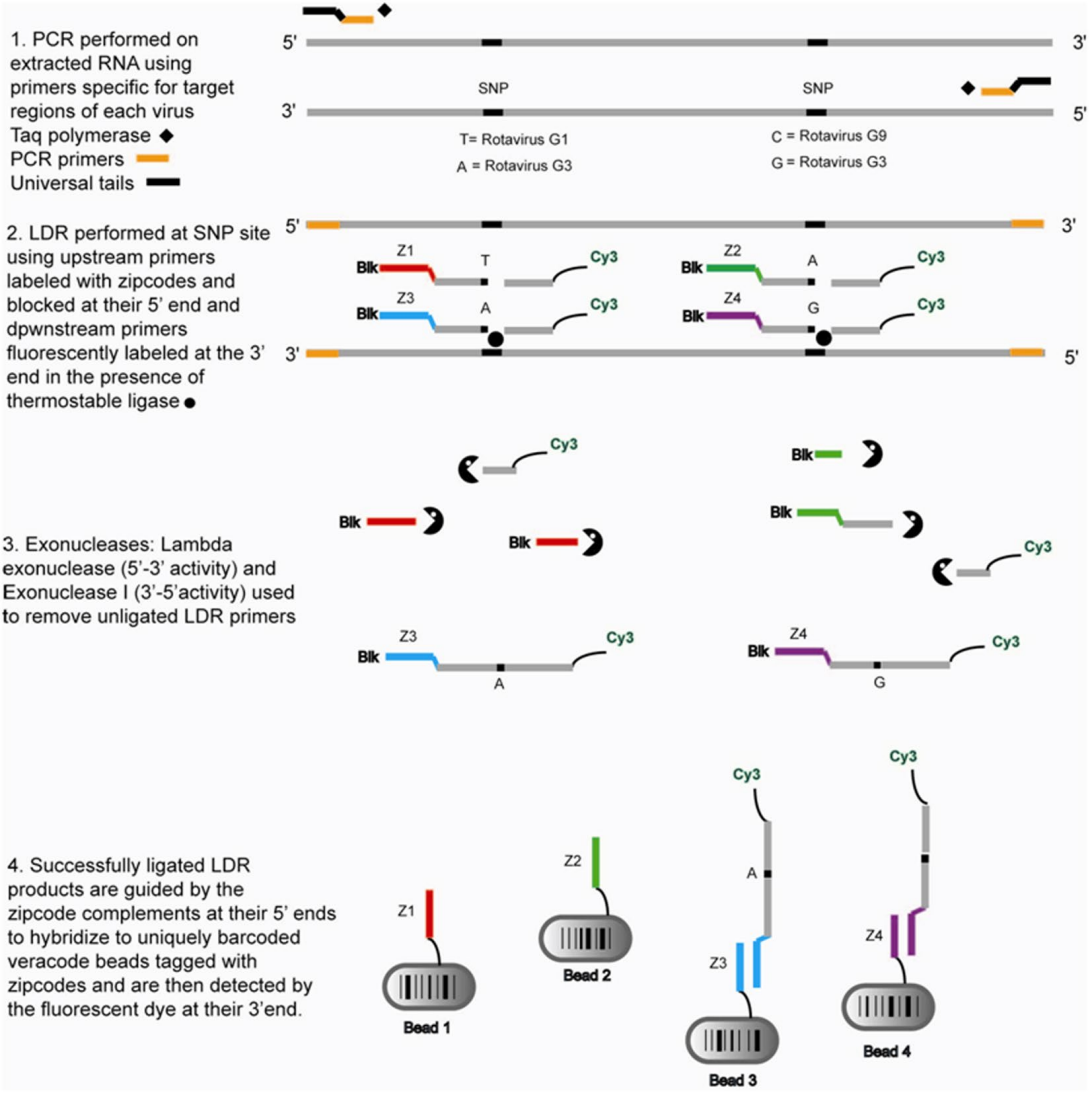
Schematic of the PCR/LDR assay followed by hybridization to the VeraCode™ micro-beads and detection using the BeadXpress platform (Illumina Inc., San Diego, CA). Virus-specific PCR primer pairs are used to amplify distinct genetic targets (one for Sapovirus and 2 each for Rotavirus and Norovirus) in a multiplex reaction. Each PCR amplicon ranging in size from 300 to 500 base pairs is then subjected to LDR using primer pairs to identify SNPs at multiple locations along the amplicon. This allows the identification of the viruses and the differentiation of the P and G genotypes of rotavirus as well as genogroup I and II of norovirus. At any given SNP, the allele specific upstream LDR primers are designed to ligate to the downstream primer only if there is a perfect match at the junction point. The upstream LDR primers bear zipcode complement sequences and amino blocking groups on the 5′-end, while the downstream LDR primers have a Cy3 fluorescent label at the 3′end. Ligation of the LDR primers results in fluorescently labeled products that are subsequently hybridized to zipcode addresses attached to VeraCode™ micro-beads. Each micro-bead contains a unique barcode that can be scanned by the BeadXpress reader. Detection of the LDR products hybridized to the specific micro-beads is accomplished by the BeadXpress reader that identifies the micro-beads by their barcode and the scores the associated fluorescent signal thus identifying the pathogen.

For the detection of the PCR amplicons, a subsequent LDR reaction was set up with up to 3 LDR primer pairs for each amplicon (132 total LDR primers in each reaction). The LDR primers were designed such that they distinguished SNPs within the PCR amplicons (Fig. 2). For example, the LDR primers for RoV were designed such that they targeted polymorphisms within the VP7 and VP4 regions to enable genotype discrimination. We also included LDR primers to detect and distinguish rotavirus genotypes G1–6, G8-10, G12 and P[3], P[4], P[6], P[8-11] and P[14]. Rotavirus genotyping was accomplished by designing LDR primers using a combination or patterns of SNPs within the VP4 and VP7 gene amplicons to permit the identification of G- and P-types by generating a distinctive pattern of signals. Therefore, for each genotype, a combination of SNPs were chosen that produce positive signals, both unique and shared between some genotypes, such that the final pattern provided unambiguous genotype identification (Supplemental Fig. 1).

The upstream LDR primers had melting temperatures of 70 °C and were labeled at the 5′-end with a unique VeraCode™ sequence and C6 amino blocking group. The downstream LDR primers were phosphorylated at the 5′-end, fluorescently labeled at the 3′-end with Cyanine 3 (Cy3) and had Tm values in the range of 70 °C-75 °C (Fig. 2 and Supplemental Table S1B). Identification of at least two LDR signals for any gene was required for pathogen identification. Sequence variations between the genotypes of the same viral species were accommodated by the use of multiple sets of primers and the inclusion of degenerate bases when required. Primers were obtained from Integrated DNA Technologies (Coralville, IA).

### Reverse-transcription PCR (RT-PCR)/Ligase Detection Reaction Assay (LDR)

A schematic of the RT-PCR/LDR assay is shown in Fig. 2. One-step RT-PCR was carried out to amplify virus specific targets. For the reverse transcription of the double stranded RNA from rotavirus samples, 5 µl of RNA (or water as a negative control) was combined with a primer mix containing 2 pmol of each primer, denatured by heating at 95 °C for 5 min, and then snap-chilled on ice for 5 min. The RT-PCR mix (One-step RT-PCR kit, QIAGEN, Valencia, CA) containing 1X buffer, 200 µM of each dNTP, 1 µl of Qiagen One-step RT-PCR Enzyme mix, and 1 unit of Qiagen RNase Inhibitor was added to a final volume of 25 µl. The thermal profile included incubation at 50 °C for 50 min for reverse transcription, followed by incubation at 95 °C for 15 min to inactivate the reverse transcriptase enzyme. Following reverse-transcription, PCR amplification of target gene sequences was achieved by thermocycling using the following parameters: 40 cycles (95 °C for 15 sec, 60 °C for 1 min and 72 °C for 1 min), a final extension at 72 °C for 7 min followed by heating at 99.9 °C for 30 min to destroy the polymerase before being held indefinitely at 4 °C.

LDR reactions were carried out as previously described^36,61,62^. LDR primers were kinased in groups for optimal results and then mixed in equal proportions to obtain a final concentration of 250 fmol/LDR reaction. The LDR products were treated with 3′–5′ and 5′–3′ exonucleases to destroy unligated LDR primers. Exonuclease reactions were performed in a total volume of 30 µl containing 10 units of Exonuclease I, 2.5 units of Lambda Exonuclease, 1X exonuclease I buffer, and 20 µl of LDR products. Samples were incubated at 37 °C for 60 min for the exonuclease reaction and then heated at 80 °C for 10 min to inactivate the enzymes.

VeraCode™ 48-plex pooled micro-beads (Illumina, San Diego, CA) containing a mix of 48 individual types of micro-beads, pre-pooled and aliquoted, were used according to the manufacturer’s instructions. Each micro-bead type detects the LDR product targeting a particular SNP. The micro-beads were mixed in 70% ethanol and distributed into 96-well microplates using the VeraCode™ Bead Kitting System. Following aspiration of ethanol, 150 µl of hybridization buffer, 1 × SSC (saline sodium citrate) and 0.05% Tween 20 was added to each well. Approximately 100 µl of the buffer was aspirated using the 8-pin aspirator manifold, and 30 µl of the LDR products was added to each well. Hybridization was carried out at 42 °C for 1 h in the dark with agitation (1000 rpm). Hybridization products were scanned using the BeadXpress Reader System with the default settings.

### Data analysis

**Figure 3:**
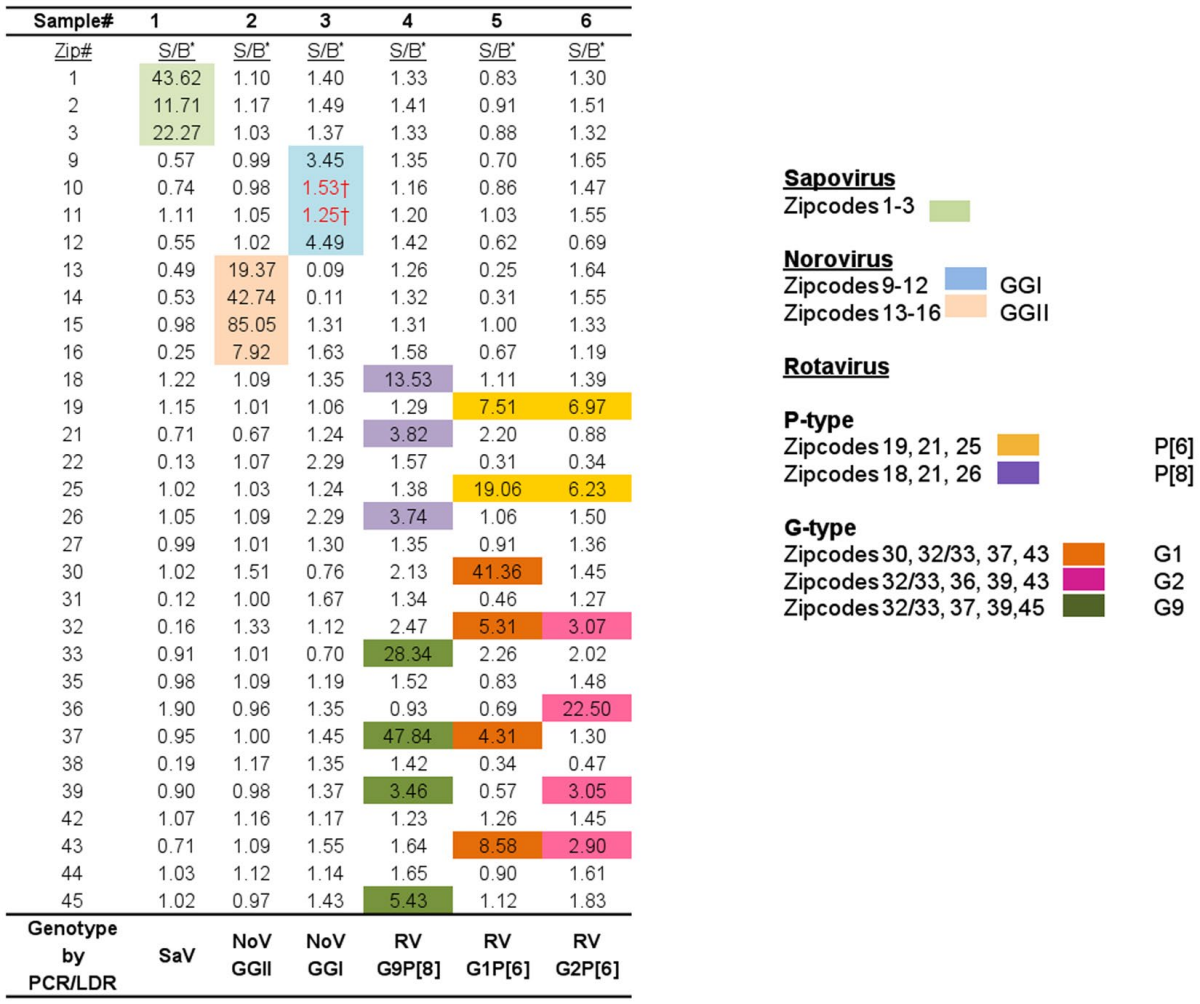
Scheme for the detection, identification, and genotyping of enteric viruses. A pattern of positive zipcode signals are used to detect and identify viruses as well as determine the genotypes of noroviruses and rotaviruses. Representative patterns of zipcode signals from 6 samples and their interpretation are shown. The normalized signal to background fluorescent signal (S/B) for each zipcode target for a given sample is indicated. An S/B value >3 fold higher than the median signal of negative controls for a given zipcode target was defined as a positive signal. The assay has built-in redundancy with at least 3 zipcode addresses assigned to identify each virus. Only 2 (for calicviruses) or 3 (for rotaviruses) positive signals are required for the identification of a virus or genotype. For example, sample 3 was identified as norovirus GGII based on 2 positive zipcode signals (9 and 12) out of the 4 designated zipcode addresses for norovirus (9, 10, 11 and 12). Similarly, samples 5 and 6 were determined as rotavirus P[6] based on a unique zipcode signature of 2 out of 3 zipcode addresses assigned (See Supplementary Fig. 1 for details of genotype determination of rotavirus). ^†^Designated zipcodes did not provide positive signals as indicated in red; however, identification was achieved successfully since the criteria of 2 out of 4 positive zipcodes was met.

Signal intensity data obtained from the BeadXpress Reader System were normalized using the median signal values of the negative controls. Signal intensities ≥3-fold higher than background were considered positive. All samples were tested by the PCR/LDR assay in duplicate and definitive identification was made when signal intensities were positive in both replicates. The scheme for the multiplexed detection, identification and genotyping of the enteric viruses included in the assay is depicted in Fig. 3.

### Rotavirus Genotype Determination by Conventional RT-PCR

To confirm the genotyping results of PCR/LDR, clinical samples were tested using the RT-PCR method as described previously^22^. The VP7 and VP4 genes were amplified using consensus primers VP7F/VP7R and Con3/Con2, respectively. Genotyping was then performed using semi-nested PCR with a combination of genotype specific primers^22^. The PCR products were resolved by electrophoresis on 2% agarose gels containing ethidium bromide and the genotypes determined by comparing the band sizes of the amplicons against a 100 bp molecular weight marker (New England Biolabs, Ipswich, MA).

### Nucleotide sequencing and phylogenetic analysis

Discrepancies in genotype identification were resolved by sequencing. Briefly, the PCR amplicons were purified using a QIAQuick PCR purification kit (Qiagen, Valencia, CA) according to the manufacturer’s instructions and were inspected for purity by electrophoresis on 2% agarose gels as well as by measuring the absorbance at 260 and 280 nm. The purified PCR products were adjusted to concentrations of 3–5 ng/µl and direct sequencing was performed at Macrogen, Inc. (Rockville, MD) using the PCR primers.

Sequence data were edited using the Lasergene software program Seqman II (DNASTAR, Inc. Madison, WI) then searched against the Genbank database using the BLASTn protocol at the National Center for Biotechnology (NCBI) website (http://www.ncbi.nlm.nih.gov/BLAST). Phylogenetic analysis was performed using the MEGA software version 5.0^63^. Genetic distances were calculated using the Kimura 2 parameter^64^, and the phylogenetic tree was constructed by the neighbor-joining method.

### Data Availability

All data generated or analyzed during this study are included in this article and its Supplementary Information files.

## Electronic supplementary material

Supplementary File
