# Supplementary material for: A Multiplex PCR/LDR Assay for Viral Agents of Diarrhea with the Capacity to Genotype Rotavirus
Source: Sci Rep. 2018 Sep 4;8:13215. doi: 10.1038/s41598-018-30301-3 (PMC6123451; doi:10.1038/s41598-018-30301-3)
Supplement: Supplementary file 1 — Supplementary File [file 41598_2018_30301_MOESM1_ESM.docx]

**Title: A Multiplex PCR/LDR Assay for Viral Agents of Diarrhea with the Capacity to Genotype Rotavirus**

Aashiq H. Mirza, Sanchita Das, Maneesh R. Pingle, Mark S. Rundell, George Armah, Ben Gyan, Richard L. Hodinka, Davise H. Larone, Eric D. Spitzer, Francis Barany and Linnie M. Golightly

| **Rotavirus P-typing** | | | | | | | | | |
| --- | --- | --- | --- | --- | --- | --- | --- | --- | --- |
|  | P3 | P4 | P6 | P8 | P9 | P10 | P11 | P14 |  |
| Zip 17 |  |  |  |  |  |  |  |  | Zip 17 |
| Zip 18 |  |  |  |  |  |  |  |  | Zip 18 |
| Zip 19 |  |  |  |  |  |  |  |  | Zip 19 |
| Zip 20 |  |  |  |  |  |  |  |  | Zip 20 |
| Zip 21 |  |  |  |  |  |  |  |  | Zip 21 |
| Zip 22 |  |  |  |  |  |  |  |  | Zip 22 |
| Zip 23 |  |  |  |  |  |  |  |  | Zip 23 |
| Zip 24 |  |  |  |  |  |  |  |  | Zip 24 |
| Zip 25 |  |  |  |  |  |  |  |  | Zip 25 |
| Zip 26 |  |  |  |  |  |  |  |  | Zip 26 |
| Zip 27 |  |  |  |  |  |  |  |  | Zip 27 |
| Zip 28 |  |  |  |  |  |  |  |  | Zip 28 |
| Zip 29 |  |  |  |  |  |  |  |  | Zip 29 |

| **Rotavirus G-typing** | | | | | | | | | | | |
| --- | --- | --- | --- | --- | --- | --- | --- | --- | --- | --- | --- |
|  | G1 | G2 | G3 | G4 | G5 | G6 | G8 | G9 | G10 | G12 |  |
| Zip 30 |  |  |  |  |  |  |  |  |  |  | Zip 30 |
| Zip 31 |  |  |  |  |  |  |  |  |  |  | Zip 31 |
| Zip 32 |  |  |  |  |  |  |  |  |  |  | Zip 32 |
| Zip 33 |  |  |  |  |  |  |  |  |  |  | Zip 33 |
| Zip 34 |  |  |  |  |  |  |  |  |  |  | Zip 34 |
| Zip 35 |  |  |  |  |  |  |  |  |  |  | Zip 35 |
| Zip 36 |  |  |  |  |  |  |  |  |  |  | Zip 36 |
| Zip 37 |  |  |  |  |  |  |  |  |  |  | Zip 37 |
| Zip 38 |  |  |  |  |  |  |  |  |  |  | Zip 38 |
| Zip 39 |  |  |  |  |  |  |  |  |  |  | Zip 39 |
| Zip 40 |  |  |  |  |  |  |  |  |  |  | Zip 40 |
| Zip 41 |  |  |  |  |  |  |  |  |  |  | Zip 41 |
| Zip 42 |  |  |  |  |  |  |  |  |  |  | Zip 42 |
| Zip 43 |  |  |  |  |  |  |  |  |  |  | Zip 43 |
| Zip 44 |  |  |  |  |  |  |  |  |  |  | Zip 44 |
| Zip 45 |  |  |  |  |  |  |  |  |  |  | Zip 45 |

**Supplemental Figure 1**. Schematic of zipcode patterns used to determine rotavirus G- and P-types.

Rows indicate the zipcode number and columns indicate associated G- and P-types. A positive signal for a unique patterns was essential for P-type determination. Unique P-type patterns are indicated by color codes. For example, two positive signals, zip 18 and 25would be adequate to call a specimen P[4] though upto 4 zipcodes were assigned to each P-type. For G-type determination, a combination strategy of essential (unique) and non-essential (shared) zipcode combinations was used. For each G-type unique zipcode addresses are designated using colored boxes (one for each zipcode) while shared zipcode addresses are shown as tan boxes.A combination of at least 2 unique and one shared zipcode were required to identify a particular G-type. If sufficient zipcodes were not positive for genotype identification, a positive rotavirus identification can be called if 2 zipcodes are positive for any/either target. See Table 2 and text for further details.

**Title: A Multiplex PCR/LDR Assay for Viral Agents of Diarrhea with the Capacity to Genotype Rotavirus**

Aashiq H.Mirza, Sanchita Das, Maneesh R. Pingle, Mark S. Rundell, George Armah, Ben Gyan, Richard L. Hodinka, Davise H. Larone, Eric D. Spitzer, Francis Barany and Linnie M. Golightly

| Organism | PCR primer | Sequence (5´to 3´) | Gene |
| --- | --- | --- | --- |
| Rotavirus  Amplicon1 | Rota-G-PCR-F1 | GCC AAC TAC CGC AAC ACG TAT GGT ATT GAA TAT ACC ACA DTT CTR AYC | VP7 |
|  | Rota-G-PCR-F2 | GCC AAC TAC CGC AAC ACG TAT GGT ATT GAA TAT ACC ACA RTT YTA ACY T |  |
|  | Rota-G-PCR-F3 | GCC AAC TAC CGC AAC ACG TAT GGT ATT GAA TAT ACC ACA AYT CTA AYY T |  |
|  | Rota-G-PCR-F4 | GCC AAC TAC CGC AAC ACG TAT GGT ATT GAA TAT ACC ACA RTT CTR ACC |  |
|  | Rota-G-PCR-F5 | GCC AAC TAC CGC AAC ACG TAT GGT ATT GAA TAT ACC GCA GTT CTA WTB T |  |
|  | Rota-G-PCR-F6 | GCC AAC TAC CGC AAC ACG TAT GGT ATT GAT TAT ACC TCA GTA TTA GTC T |  |
|  | Rota-G-PCR-R1 | CCA ACT ACC GCA ACC AGTT ATA TCC ATT GGR TTR CAT AAC CAY TC |  |
|  | Rota-G-PCR-R2 | CCA ACT ACC GCA ACC AGTA ATR TCC ATM GGA TTA CAT AAC CAY TC |  |
|  | Rota-G-PCR-R3 | CCA ACT ACC GCA ACC ATGT TAT RTC CAT TGG RTT ACA TAA CCA TTC |  |
|  | Rota-G-PCR-R4 | CCA ACT ACC GCA ACC AAGT WAT WTC CAT TGG RTT ACA TAA CCA YTC |  |
|  | Rota-G-PCR-R5 | CCA ACT ACC GCA ACC AGTM ATT TCC ATY GGA TTA CAT AAC CAY TC |  |
|  | Rota-G-PCR-R6 | CCA ACT ACC GCA ACC AGTK ATA TCC ACT YGG RTT ACA TAA CCA TTC |  |
|  | Rota-G-PCR-R7 | CCA ACT ACC GCA ACC AGTA ATA TCC ACT WGG RTT ACA TAA CCA TTC |  |
|  | Rota-G-PCR-R8 | CCA ACT ACC GCA ACC ATGT TAT ATC CAT TGG ATT ACA AAG CCA CTC |  |
|  | Rota-G-PCR-R9 | CCA ACT ACC GCA ACC ATGA TAT ATC CAT HGG ATT RCA YAG CCA |  |
| Rotavirus  Amplicon2 | Rota-P-PCR-F1 | GCC AAC TAC CGC AAC ACG GCT ATA AAA TGS GTT CGC TCA TTT AYA G | VP4 |
|  | Rota-P-PCR-F2 | GCC AAC TAC CGC AAC ACG GCT ATA AAA TGG CTT CTC TRA THT AYA G |  |
|  | Rota-P-PCR-F3 | GCC AAC TAC CGC AAC ACG GCT ATA AAA TGG CTW SSC TCA TTT ATA G |  |
|  | Rota-P-PCR-F4 | GCC AAC TAC CGC AAC ACG GCT ATA AAA TGG CTT CTT TAA TTT ATA G |  |
|  | Rota-P-PCR-F5 | GCC AAC TAC CGC AAC ACG GGT TTA TAA TGG CTT CGC TAA TAT ACA G |  |
|  | Rota-P-PCR-R1 | CCA ACT ACC GCA ACC ACRT CAA YAR AYC TCC ATT GCG TYT GTG |  |
|  | Rota-P-PCR-R2 | CCA ACT ACC GCA ACC ACAT YAA ATC TAT ARA CTT CCA TTT YGT ATT TG |  |
|  | Rota-P-PCR-R3 | CCA ACT ACC GCA ACC ACTA AAC ATT TCT AAA AAC TTC CAT TTA TCT G |  |
|  | Rota-P-PCR-R4 | CCA ACT ACC GCA ACC AGAT AAA TAG TAT GAA TTT CCA AAG AGT ACT TG |  |
|  | Rota-P-PCR-R5 | CCA ACT ACC GCA ACC ACGA ACC CCA ATA ATT TGA TAT ATT TCC A |  |
|  | Rota-P-PCR-R6 | CCA ACT ACC GCA ACC AGYG TTT CGC CAT GAA AYG TCC ATA C |  |
|  | Rota-P-PCR-R7 | CCA ACT ACC GCA ACC AGTT AAA GTA CGC TTA TGT TGA AAT TC |  |
|  | Rota-P-PCR-R8 | CCA ACT ACC GCA ACC AATA ATA TAA ATT TCC ACG AAG TAC TTG AAT C |  |
| Hepatitis A virus Amplicon 1 | HepA-PCR-A1-F1a | GCC AAC TAC CGC AAC ACA TGG ATG TWT CGG GTG TRC ARG C | RNA polymerase |
|  | HepA-PCR-A1-F1b | GCC AAC TAC CGC AAC A**C**A TGG ATA TTT CGG GTG TCC AAG C |  |
|  | HepA-PCR-A1-R1 | CCA ACT ACC GCA ACC AGG AGT RAA CCA RGC CAT RCC ATC |  |
| Hepatitis A virus Amplicon 2 | HepA-PCR-A2-F1 | GCC AAC TAC CGC AAC ACC CAT GGG TDG ARA AGG AGT CWG CT | RNA polymerase |
|  | HepA-PCR-A2-R1a | CCA ACT ACC GCA ACC ATC ATC CAC YGA CGA YTC CAA RTC TCA |  |
|  | HepA-PCR-A2-R1b | CCA ACT ACC GCA ACC AGG ATC ATC AAC TGA GGA TTC AAG ATC |  |
| Norovirus Amplicon 1 | NoVG1-PCR-A1-F2 | GCC AAC TAC CGC AAC ACG ATG ATG AGA TMG TGT CAA CWG ACR TA | ORF1-ORF2 junction |
|  | NoV-G1-PCR-A1-R2a | CCA ACT ACC GCA ACC AAY CGC ATC CAR CGG AAC ATG |  |
|  | NoV-G1-PCR-A1-R2b | CCA ACT ACC GCA ACC AAG CGC ATC CAR CGG AAC ATG |  |
|  | NoroG2-PCR-A1-F1 | GCC AAC TAC CGC AAC ACG GYA AGA ARC ACA CRG CAT TCT C |  |
|  | NoroG2-PCR-A1-F2 | GCC AAC TAC CGC AAC ACG GCA AGA AAC ACA CMG CHT TYT C |  |
|  | NoroG2-PCR-A1-R1 | CCA ACT ACC GCA ACC ACA CTY CTR TCA TCG TCR GCC CAC AG |  |
|  | NoroG2-PCR-A1-R2 | CCA ACT ACC GCA ACC ACA CTY CTG TCR TCA TCA GCC CAC AG |  |
| Norovirus Amplicon 2 | NoroG1-G2-PCR-A2-F1 | GCC AAC TAC CGC AAC ACC TGA TGG GTC YRC AGC CAA CCT | ORF1-ORF2 junction |
|  | NoroG1-G2-PCR-A2-R1 | CCA ACT ACC GCA ACC ATC AGT WGG RAA GTT TGG WGG GAC TG |  |
| Sapovirus  Amplicon1 | Sapo-A1-PCR-F1 | GCC AAC TAC CGC AAC ACA TGG AGG GCA ATG GCT CCM R |  |
|  | Sapo-A1-PCR-F2 | GCC AAC TAC CGC AAC ACG AGG GCA ATG GCC TMC CCCA | VP1 capsid protein |
|  | Sapo-A1-PCR-R1 | CCA ACT ACC GCA ACC AGT GCC TGT GGC CTG TCG AGT GTT |  |
|  | Sapo-A1-PCR-R2 | CCA ACT ACC GCA ACC AAC ATC CCR GAG AGG TGA GCA GTG TAC |  |
|  | Sapo-A1-PCR-R3 | CCA ACT ACC GCA ACC AAC ATS CCC GAG AGG TGY GAA GTG TAC |  |
|  | Sapo-A1-PCR-R4 | CCA ACT ACC GCA ACC AAC ATG CCT GAA AGA TGG GAT GTG TAT |  |
|  | Sapo-A1-PCR-R5 | CCA ACT ACC GCA ACC AGT CAG GAT GTA RAG ACA CAG CTG TGA G |  |

**Supplemental Table S1A**. PCR primers used for detection of agents of viral diarrhea.

Multiple primers were designed for each amplicon for the different viruses and multiple primers for the same virus were required to accommodate sequence variations and these are then differentiated by the numbers and letters. The underlined nucleotides indicate the sequence of the universal tails.

| Upstream primers | | Downstream primers | |
| --- | --- | --- | --- |
| Primer name | Sequence (5’-3’) | Primer name | Sequence (5’-3’) |
| Rota-G-LDR-UP-1a | Blk-GCCATATACTCTCCAGTGCGTG  GTTTCTYACRAAAGGTTGGCCAACAGG | Rota-G-LDR-DP-1a | ATCTGTTTACTTCAAAGAGTACTCAAGTATTGT |
| Rota-G-LDR-UP-1b | Blk-ATGTTGAAGCCAAGCCGCTGTG  ACAGAYATAGCAGCKTTTTCAGTWGACC | Rota-G-LDR-DP-1b | ATCAGTATATTTTAAAGAGTAYTCAAATATYGT |
|  |  | Rota-G-LDR-DP-1c | CTCAGTTGTATTGTGATTACAATTTAGTCCT |
|  |  | Rota-G-LDR-DP-1d | CACAACTATACTGCGATTACAATATAGTTCT |
| Rota-G-LDR-UP-2a | Blk-GTTTGCCAGCCAGTGGAACGAC  ACATTATGTYTRTATTATCCAACTGAAGC | Rota-G-LDR-DP-2a | AAGTAMTCAAATCARTGATGGTGAMTGGA |
| Rota-G-LDR-UP-2b | Blk-GTTTGCCAGCCAGTGGAACGAC  ACGCTATGTYTRTAYTATCCARCAGAAGC | Rota-G-LDR-DP-2b | WAAAAATGAGATTKCAGATRATGAATG |
| Rota-G-LDR-UP-2c | Blk-GTTTGCCAGCCAGTGGAACGAC  ACGCTRTGYTTATAYTATCCAGCAGAAGC | Rota-G-LDR-DP-2c | AGCAACTGAAATAAATGATRATTCATGGA |
| Rota-G-LDR-UP-2d | Blk-GTTTGCCAGCCAGTGGAACGAC  ACTTCGACTTTRTGTTTRTAYTAYCCAACTGAAGC | Rota-G-LDR-DP-2d | TGCRGCWGAGATAAAYGATAATTCATGGA |
| Rota-G-LDR-UP-2e | Blk-GTTTGCCAGCCAGTGGAACGAC  ACGTCTACTYTATGCCTRTAYTATCCTACTGAGGC | Rota-G-LDR-DP-2e | GGCTACTGAAATTGCRGATARTAAGT |
| Rota-G-LDR-UP-2f | Blk-TCAGCGGTTAGTACGGTCACATG  ACATCAACWCTGTGCCTATATTATCCAACTGARGC | Rota-G-LDR-DP-2f | GGCTACTGAAATTGCAGACGCCAAGT |
| Rota-G-LDR-UP-2g | Blk-TCAGCGGTTAGTACGGTCACATG  TTATGYTTRTATTAYCCGAATGAAGC | Rota-G-LDR-DP-2g | ATCRAAYGARATMGCTGAYACSGAATGGA |
| Rota-G-LDR-UP-2h | Blk-TCAGCGGTTAGTACGGTCACATG  CTCTGTGTTTGTATTAYCCAGTCGAAGC | Rota-G-LDR-DP-2h | RTCAACTCAAATTGGAGATACGGAAT |
| Rota-G-LDR-UP-2i | Blk-TCAGCGGTTAGTACGGTCACATG  CCCTTTGCCTATATTATCCAACCGAAGC | Rota-G-LDR-DP-2i | TAGRACAGAAATAAATGATAATGAGTGGAYRART |
| Rota-G-LDR-UP-2j | Blk-TCAGCGGTTAGTACGGTCACATG  AACGCTATGYTTATATTMTCCTACAGAAGC | Rota-G-LDR-DP-2j | AGCTCCAACTCAAATTAGTGACACTGA |
| Rota-G-LDR-UP-2k | Blk-TCAGCGGTTAGTACGGTCACATG  CGTTATGTTTRTATTACCCAACRGAAGC | Rota-G-LDR-DP-2k | AGCAAGTACTCAAATCAATGATGGTGA |
| Rota-G-LDR-UP-2l | Blk-TCTAGCAACGGGTAATTGTCGGC  CTTCAACWTTATGTYTRTATTATCCAWCWGA | Rota-G-LDR-DP-2l | CACGACTGAAATAACTGAYCCYGAYT |
| Rota-G-LDR-UP-2m | Blk-TCTCAATGACACGACGGCAACTG  CCACTTTATGYTTATATTATCCAAGTTYAGT |  |  |
| Rota-G-LDR-UP-2n | Blk-TCTCAATGACACGACGGCAACTG  TCATGGAAAGAAACATTGTCACAACTGT |  |  |
| Rota-G-LDR-UP-2o | Blk-TCTCAATGACACGACGGCAACTG  TCRTGGAAAGAYACWTTRTCRCARTTAT |  |  |
| Rota-G-LDR-UP-3a | Blk-CTGGACTTCAACGAACGAGCATC  TCACTAGACGCTGTATAYACRAATTCRACYA | Rota-G-LDR-DP-3a | GTGRAGARYCATTTYTAACTTCRACGCT |
| Rota-G-LDR-UP-3b | Blk-CTGGACTTCAACGAACGAGCATC  TGGATACACCATATAYGAAYTCAACRA | Rota-G-LDR-DP-3b | GTGGAGAAACATTTCTAACTTCGACRCT |
| Rota-G-LDR-UP-3c | Blk-CTGGACTTCAACGAACGAGCATC  GACKCTGTGTCTRTATTATCCAGTAGA | Rota-G-LDR-DP-3c | TGAGTGARACGTTTTTAACTTCYACATTA |
| Rota-G-LDR-UP-3d | Blk-CTGGACTTCAACGAACGAGCATC  AACGCTMTGTYTKTATTATCCAACAGA | Rota-G-LDR-DP-3d | GCWGARACAGARATWGCTGAYAGTT |
| Rota-G-LDR-UP-3e | Blk-CTGGACTTCAACGAACGAGCATC  AACATTATGCTTGTATTATCCAGTAGA | Rota-G-LDR-DP-3e | AGGRYGARCCATTCCTAACGTCAACGT |
| Rota-G-LDR-UP-3f | Blk-CTGGACTTCAACGAACGAGCATC  TGGATCAATGGATGTATYATATGCAAATACTACMA | Rota-G-LDR-DP-3f | RAGAAGRAGTRTTYCTAACWTCYACATTATGT |
| Rota-G-LDR-UP-3g | Blk-AACCTTTGCACCGTTGGAATGGC  ATCAATGGAYACTGTATACDCYAAYTCTACTC | Rota-G-LDR-DP-3g | AGGAAGAGACGCTCCTCACATCCAC |
| Rota-G-LDR-UP-3h | Blk-AACCTTTGCACCGTTGGAATGGC  ATAYTGCATACGCTAATTCWACRC | Rota-G-LDR-DP-3h | GRGAGGAAGTRTTCCTAACTTCGAC |
| Rota-G-LDR-UP-3i | Blk-AACCTTTGCACCGTTGGAATGGC  ACATACTATATACGAACTCAACRC | Rota-G-LDR-DP-3i | AAGAAGAAACTTTYCTCACWTCWAC |
| Rota-G-LDR-UP-3j | Blk-AACCTTTGCACCGTTGGAATGGC  ATCTATGGATACAGCATAYRCTAACTYAACKC | Rota-G-LDR-DP-3j | AAGAAGAGACATTCCTTACGTCTAC |
| Rota-G-LDR-UP-3k | Blk-AACCTTTGCACCGTTGGAATGGC  TCCATGGATRCYRCATAYGCAAATTCAWCAC | Rota-G-LDR-DP-3k | MARACAATAATTTTTTATCTTCAACTTTAT |
| Rota-G-LDR-UP-3l | Blk-AACCTTTGCACCGTTGGAATGGC  CTATGGATACCGCATATGYAAACWTACRC | Rota-G-LDR-DP-3l | AAGAAGGAATATTTCTAACATCCACATTAT |
|  |  | Rota-G-LDR-DP-3m | AAAACAATAATAATTTTTTATCTTCAACTTTAT |
| Rota-G-LDR-UP-4a | Blk-GCCTACCGATTGTAAGACCATGC  AGTAGTGGTRATACTGACCACCGTGACAAATG | Rota-G-LDR-DP-4a | AAAGTGAATCTTTTTTAACATCAACTCTGTGTT |
| Rota-G-LDR-UP-4b | Blk-GCCTACCGATTGTAAGACCATGC  CGTTGTAGTCATTATGACCATCATTGTGAACG | Rota-G-LDR-DP-4b | AGAACGAGCCGTTTTTGACATCGACCCTTTGCC |
| Rota-G-LDR-UP-4c | Blk-GCCTACCGATTGTAAGACCATGC  TACTTATAGTCACAATTACTTCAATCTTTGTCAATG | Rota-G-LDR-DP-4c | ARCAAGAAACATTTTTGACTTCAACGC |
| Rota-G-LDR-UP-4d | Blk-GCCTACCGATTGTAAGACCATGC  TACTTATAGTCATGATCATTTCAGTCGTTGTTAACG | Rota-G-LDR-DP-4d | AACAAGAGAATTYTATGACTTCCACTTTAT |
| Rota-G-LDR-UP-4e | Blk-GCCTACCGATTGTAAGACCATGC  AATAGTTGTYGTCRTRCTGCCATTTATTAAAG | Rota-G-LDR-DP-4e | CGCAAAATTATGGARTWAATYTRCCCATTA |
| Rota-G-LDR-UP-4f | Blk-TGGATCGGGTCTAAGCTCTATGG  CATYGCTCTRATRTCACCATTTGTRAGGA | Rota-G-LDR-DP-4f | CACARAAYTACGGTATTAACTTACCAAT |
| Rota-G-LDR-UP-4g | Blk-TGGATCGGGTCTAAGCTCTATGG  CATTGYTMTGATATCRCCATTTGTRAGRA | Rota-G-LDR-DP-4g | CTCAAAATTATGGAAWAAATCTTCCAATAACA |
| Rota-G-LDR-UP-4h | Blk-TGGATCGGGTCTAAGCTCTATGG  YATTGCTTTRATATCACCATTTGTRAGGA | Rota-G-LDR-DP-4h | RGCTCAGAAYTATGGAATYAATTTGCC |
| Rota-G-LDR-UP-4i | Blk-TGGATCGGGTCTAAGCTCTATGG  TTATAGTTTTGCTTGCACCAYTTATTAA | Rota-G-LDR-DP-4i | CACAGAAYTATGGYATWAAYYTGCCAA |
| Rota-G-LDR-UP-4j | Blk-TGGATCGGGTCTAAGCTCTATGG  ACTTATTGTTATTGCATCAYCTTTTGTTAARA | Rota-G-LDR-DP-4j | CRCARAAYTATGGYATWAATTTACCGA |
| Rota-G-LDR-UP-4k | Blk-TGATGTCAGATGCCGTTCAGTCG  TTRTTGTAATTGTCACACCATTYGTAAAYT | Rota-G-LDR-DP-4k | MTCAGAAYTATGGRCTTAAYWTACCAATAACA |
| Rota-G-LDR-UP-4l | Blk-TGATGTCAGATGCCGTTCAGTCG  TTGTTGTTGTTRTCGCACCATTTGTRAATT | Rota-G-LDR-DP-4l | CDCAAAATTATGGWATWAATCTWCCRA |
| Rota-G-LDR-UP-4m | Blk-GCTCCACACGTACATGGTAATCC  TRTAGCATTATTTGYHTTAACTAAAG | Rota-G-LDR-DP-4m | CKCAAAATTATGGAATWAATCTACCAA |
| Rota-G-LDR-UP-4n | Blk-GCTCCACACGTACATGGTAATCC  TGTARCATTATTTGCCTTRACRAGAG | Rota-G-LDR-DP-4n | CRCAAAATTATGGAATAAATTTGCCAAY |
| Rota-G-LDR-UP-4o | Blk-GCTCCACACGTACATGGTAATCC  TGTAGCATTATTTGYCTTAACTAARG | Rota-G-LDR-DP-4o | CTCAGAACTATGGACTTAATATACCAATAAC |
| Rota-G-LDR-UP-4r | Blk-ATGCGAAGAGTATGGACCTCACC  TTTTATAATTGTGATATTGTCACCATTTCTCAGAG | Rota-G-LDR-DP-4p | CATYATATAYAGATTTTTRTTAATTWYTGTAGCAT |
| Rota-G-LDR-UP-4s | Blk-ATGCGAAGAGTATGGACCTCACC  TTTARTTATAGTTATAYTRTCACCACTCCTTAATG | Rota-G-LDR-DP-4q | CATTATATATAGATYTTTATTRATTTMTGTAGCAT |
| Rota-G-LDR-UP-4t | Blk-ATGCGAAGAGTATGGACCTCACC  TTTCATCATAGTTATYTTRTCACCATTGCTAAARG | Rota-G-LDR-DP-4r | CATAATTTTYAGATTTTTATTACTCATCGCTCTRATRTCAC |
| Rota-G-LDR-UP-4U | Blk-ATGCGAAGAGTATGGACCTCACC  CTTTATTRTAGTCATTCTGTCACCATTGTTGAAAG | Rota-G-LDR-DP-4s | CATAATTTTCAGRTTTTTACTACTCATTGCTYTRATATCAC |
| Rota-G-LDR-UP-4v | Blk-ATGCGAAGAGTATGGACCTCACC  TWTCTGTAGCATTATTTGCCTTGACTAAAG | Rota-G-LDR-DP-4t | CATAATTTTYAGATTTTTACTGCTCATTGCTMTGATATCAC |
| Rota-G-LDR-UP-4x | Blk-ATGCGAAGAGTATGGACCTCACC  TGTAATTGTAGTATTATCAGTATTRKCGAATG | Rota-G-LDR-DP-4u | CATAATCTATAGATTTTTACTAGTCATTGTTCTGATATCAC |
|  |  | Rota-G-LDR-DP-4v | CATAATTTTCAGRTTTTTACTACTCATTGCTTTAATATCAC |
|  |  | Rota-G-LDR-DP-4x | TATAATTTTTAGGTTATTACTACTCATCGCTCTGATRTCAC |
|  |  | Rota-G-LDR-DP-4y | TATTATTTATAGAAAAAAATTTGTAATTGTAGTATTATCA |
| Rota-G-LDR-UP-5a | Blk-AAAGCCTCCGTGATTCATCTGCG  TATATATTAAAATCAGTRACYCRAATAATGGACTA | Rota-G-LDR-DP-5a | YATAATTTATAGGTTTCTATTAGTAGTGGTAATACTGA |
| Rota-G-LDR-UP-5b | Blk-AAAGCCTCCGTGATTCATCTGCG  TTAYATATTAAAAACTATAACTAAYACRATGGACTA | Rota-G-LDR-DP-5b | TATAATTTATAGATTCTTGTTCGTTGTAGTCATTATGA |
| Rota-G-LDR-UP-5c | Blk-AAAGCCTCCGTGATTCATCTGCG  TCTGAGGACCATAATAAAGATAATGGACTA | Rota-G-LDR-DP-5c | TATATTGTATAGATTCTTGCTATTTGTTGTAAT |
| Rota-G-LDR-UP-5d | Blk-AAAGCCTCCGTGATTCATCTGCG  TATATATTAAAATCARTAACRAGRATAATGGACTA | Rota-G-LDR-DP-5d | YATACTCTATCGTTTTTTGCTTTTTRTTGTKAT |
| Rota-G-LDR-UP-5e | Blk-ATTATATCACCCTGGTCCGTCTGG  TAAAATCARTRACMWGRRTTATGGACTA | Rota-G-LDR-DP-5e | CATATTGTATCGATTCTTGTTTTTTGTTGTTGT |
| Rota-G-LDR-UP-5f | Blk-ATTATATCACCCTGGTCCGTCTGG  TAAAAWCAATAACYAGAGTRATGGACTA | Rota-G-LDR-DP-5f | CATACTCTACAGATTTYTGCTYTTTATTGTRAT |
| Rota-G-LDR-UP-5g | Blk-ATTATATCACCCTGGTCCGTCTGG  AATCAACAACAGTATAGAAAACTTGCCA | Rota-G-LDR-DP-5g | YATAATTTATAAATTTTTACTTATAGTCACAATTA |
| Rota-G-LDR-UP-5h | Blk-ATTATATCACCCTGGTCCGTCTGG  TACATACTTAAAAGTATAACTAGGATGATGGACTA | Rota-G-LDR-DP-5h | TATAATTTATAGATTTCTTTTTATAATTGTGATATTGTCAC |
| Rota-G-LDR-UP-5i | Blk-ATTATATCACCCTGGTCCGTCTGG  TATATATTAAAGAGYATAACTAGAATGATGGACYA | Rota-G-LDR-DP-5i | TATTATTTACAGATTTCTTTTARTTATAGTTATAYTRTCAC |
| Rota-G-LDR-UP-5j | Blk-CAGGCACGAATTGTTTAGGGTCAA  CRTACTYAAATCMTTAACTAGAATAATGGACTT | Rota-G-LDR-DP-5j | TGTCATTTATAGATTTCTTTTCGTTATAGTCGTCCTGTCAC |
| Rota-G-LDR-UP-5k | Blk-CAGGCACGAATTGTTTAGGGTCAA  TATRYTKAAATCTTTGACTAGAATGATGGACTT | Rota-G-LDR-DP-5k | TATCATTTACAGGTTTCTCTTTATTGTAGTCATTCTGTCAC |
| Rota-G-LDR-UP-5l | Blk-CAGGCACGAATTGTTTAGGGTCAA  TATACTAAAATCYTTRACYAGAATGATGGACTT | Rota-G-LDR-DP-5l | TATMATCTATAGATTCCTTTTYATCATAGTYATCTTGTCAC |
| Rota-G-LDR-UP-5m | Blk-CAGGCACGAATTGTTTAGGGTCAA  TTAATTAYTTATTAAAATCASTRACTAGWGYRATGGACTT | Rota-G-LDR-DP-5m | YATTATYTACAGATTTCTTTTMATTATAGTTGTACTGTCGC |
| Rota-G-LDR-UP-5n | Blk-CAGGCACGAATTGTTTAGGGTCAA  TATATATTAAAATCAATAACTAATATAATGGACKYT | Rota-G-LDR-DP-5n | TATYATTTATAGATTTTTAYTARTTTTATAGTTT |
|  |  | Rota-G-LDR-DP-5o | TATAATTTATAGATTTCTTTTACTTATTGTTATT |
|  |  | Rota-G-LDR-DP-5p | ATCATATATCGGTTTTTACTAATAGTTGT |
| Rota-P-LDR-UP-1a | Blk-CCTACGAGTGGGTGGTCAACGA  AGATCGGGTCTACGAAAACTCAAAATA | Rota-P-DP-LDR1a | AGATCGGGTCTACGAAAACTCAAAATA |
| rota-P-LDR-UP-1b | Blk-CCTACGAGTGGGTGGTCAACGA  TTCATATACYGTAAATYTATCAGATGAGATACAAG | Rota-P-DP-LDR1b | AAATTGGARCTAAAAAAACAACTAAYGTTACT |
| rota-P-LDR-UP-1c | Blk-CCTACGAGTGGGTGGTCAACGA  ARATCGGAGCTAGRAAAACAACAAATGT | Rota-P-DP-LDR1c | ARATCGGAGCTAGRAAAACAACAAATGT |
| rota-P-LDR-UP-1d | Blk-CCTACGAGTGGGTGGTCAACGA  AAATTGGRGCTAGGAAARCAACRAACGT | Rota-P-DP-LDR1d | AAATTGGRGCTAGGAAARCAACRAACGT |
| rota-P-LDR-UP-1e | Blk- TATTCACGCAGGGTAAGGGCAC CATATTCAGTAGATTTAYATGATGAAATAGAGC | Rota-P-DP-LDR1e | AGATTGGAGCTAGAAAGACGACAAACGT |
| rota-P-LDR-UP-1f | Blk- TATTCACGCAGGGTAAGGGCAC CATATTCGGTAGACTTRCATGACGAAATAGARC | Rota-P-DP-LDR1f | ARGTTGGATCRGAGAAAACTCAAARTGT |
| rota-P-LDR-UP-1f_rep | Blk- TATTCACGCAGGGTAAGGGCAC CATATTCRGTAGAYTTGCATGATGAAATAGAAC | Rota-P-DP-LDR1f_rep | ARATTGGATCRGAGAAAACTCAAARTGT |
| rota-P-LDR-UP-1g | Blk-GCATCAACTCTTCGGTGGGCTA CATACACAGTTGAATTATCAGAYGAAATTARYA | Rota-P-DP-LDR1g | AAATTGGATCAGAAAAAACTCAAAATGTAACT |
| rota-P-LDR-UP-1h | Blk- GCATCAACTCTTCGGTGGGCTA CATACACAGTTGAACTGTCAGACGAAATTAATA | Rota-P-DP-LDR1h | CAATTGGATCAGARAAAARTCAAAAYGTGAC |
| rota-P-LDR-UP-1i | Blk- GCATCAACTCTTCGGTGGGCTA TAACTCTTATACAGTAGAYTTATCTGAYGAAATTGAGA | Rota-P-DP-LDR1i | CGATTGGATCAGARAAAAGTCARAATGTAAC |
| rota-P-LDR-UP-1k | Blk- AGCCTGTCTTAATCCGGGTGAC TAACTCATATTCAGTAGATTTATCAGATGAAATAACT | Rota-P-DP-LDR1j | WAATTGGATCAGARAAAAGTCAAAATGTRAC |
|  |  | Rota-P-DP-LDR1k | GTATYGGATCTRAAAAAAYTCAAAATGTAAC |
|  |  | Rota-P-DP-LDR1l | AATATAGGTGCAGAAAAGAAAGAGAATGTGA |
| Rota-P-LDR-UP-2a | Blk-ACTGTCCACCTAGAAGCGACGT ATTTGCACAAACTGGMTAYGCYCCAG | Rota_P_DP-LDR2a | TRAAYTGGGGTCCYGGTGAAACGAA |
| Rota-P-LDR-UP-2b | Blk-ACTGTCCACCTAGAAGCGACGT CATTTGCACARACYAGATAYGCTCCAG | Rota_P_DP-LDR2b | TYRATTGGGGACACGGAGAGATTAATGATTC |
| Rota-P-LDR-UP-2c | Blk-ACTGTCCACCTAGAAGCGACGT GTTYGCTCAAACAAATTAYGCRCCAG | Rota_P_DP-LDR2c | TTAATTGGRGWCAYGGGGAGACTAATGATTC |
| Rota-P-LDR-UP-2d | Blk-ACTGTCCACCTAGAAGCGACGT ATTTGCTCAAACAAGATATGCTCCAG | Rota_P_DP-LDR2d | TRACTTGGAGTCAYGGRGAAGTGAATGA |
| Rota-P-LDR-UP-2e | Blk-ACTGTCCACCTAGAAGCGACGT GCACAAACCGGRTATGCACCAG | Rota_P_DP-LDR2e | TYAATTGGGGYCATGGAGAGRTTAATGAT |
| Rota-P-LDR-UP-2f | Blk-ACTGTCCACCTAGAAGCGACGT TCGCACAGACTGGATATGCTCCAG | Rota_P_DP-LDR2f | TTAATTGGGGACCAGGAGAGGTRAATGAC |
| Rota-P-LDR-UP-2g | Blk-ACTGTCCACCTAGAAGCGACGT TTGCGCAGACHGGGTAYGCWCCAG | Rota_P_DP-LDR2g | TYRATTGGGGACAYGGTGAATTGTC |
| Rota-P-LDR-UP-2h | Blk-GGCTCACTGGGAACGTGCTTAT CAGGRCCRTTYGCACARACGGGRT | Rota_P_DP-LDR2h | ATGCGCCTGTYRAYTGGGGACAT |
| Rota-P-LDR-UP-2i | Blk-GGAACTCTCCTTAGATCGCCGC TTCGCTCAAAGTCAATACGCACCA | Rota_P_DP-LDR2i | GTTTCRTGGGGATCAGGAGAGAYRTTAA |
| Rota-P-LDR-UP-3a | Blk-CTACTGGGCTGTGCGGAACGAC  ATAYCAACCAACYACATTTAATCCACCAGT | Rota-P-LDR-DP-3a | AGATTATTGGATGTTACTAGCGCCTGCGA |
| Rota-P-LDR-UP-3b | Blk-TTATCTCCACACCACGAGGCGT  GGTCCTTATCAACCMACTACATTCAAACCACCYAA | Rota-P-LDR-DP-3b | TGATTATTGGYTRCTYATTAGYTCAAATAC |
| Rota-P-LDR-UP-3c | Blk-TTATCTCCACACCACGAGGCGT  TATCARCCAACAAGYTTTAAACCRCCAA | Rota-P-LDR-DP-3c | RYGATTACTGGATAYTATTDAATCCAACTAATCAAC |
| Rota-P-LDR-UP-3d | Blk-TTATCTCCACACCACGAGGCGT  TATCARCCAACAARTTTTAARCCACCAA | Rota-P-LDR-DP-3d | TGATTACTGGATACTTATTAAYTCRAATACAAATG |
| Rota-P-LDR-UP-3e | Blk-TTATCTCCACACCACGAGGCGT TGATGGTCCATATCAACCYACYTCAYTCAA | Rota-P-LDR-DP-3e | YGAYTATTGGATGYTAATTGCRCCTACYCA |
| Rota-P-LDR-UP-3f | Blk-TACGCGACAGATGGGTGATCCA CCTTATCAACCTACYACATTTACACCACCTAC | Rota-P-LDR-DP-3f | TAACTATTGGATGCTGATTGCACCAACTCAAGCGGGTA |
| Rota-P-LDR-UP-3h | Blk-TCTCGCCTGAAGGACCAGTTGC CCTATCAACMAACAAGCTTYAATCCACCAGTT | Rota-P-LDR-DP-3g | AAYTATTGGATGCTTTTAGCYCCGTTAAAT |
| Rota-P-LDRUP-3i | Blk-TCTCGCCTGAAGGACCAGTTGC CCATATACACCAGATARBTCAAATTTRCCGT | Rota-P-LDR-DP-3h | CTAACTATTGGTATTTRATCAATCCATYAAATGAT |
| Rota-P-LDR-UP-3j | Blk-TCTCGCCTGAAGGACCAGTTGC  CCATATCAACCAACYACATTTAATYTGCCWAT |  |  |
| Rota-P-LDR-UP-3k | Blk-TCTCGCCTGAAGGACCAGTTGC TCAACMAACAAGCTTYAATCCACCAGTT |  |  |
| HepA-A1-LDR-UP-1 | Blk- CTGCGGCTACGTTCGTACAGAT  GAGGATCCAGTTTTAGCAAAGAAAGTRCCT | HepA-A1-LDR-DP-1 | GAGACATTYCCWGAATTGAAACCTGGAGA |
| HepA-A1-LDR-UP-2a | Blk- AGCCTACGATCTGCGGTGCAAC  CCAGRCATACATCAGATCATATGTCYATYTACAAGTTTAT | HepA-A1-LDR-DP-2a | GGGAAGGTCTCATTTTYTGTGYACTTTTACYTTCA |
| HepA –A1-LDR-UP-2b | Blk- AGCCTACGATCTGCGGTGCAAC  CAAGACATACTTCTGATCATATGTCTATTTATAAATTTAT | HepA-A1-LDR-DP-2b | GGGTAGATCTCATTTCTTGTGCACATTTACATTTA |
| HepA-A1-LDR-UP-2c | Blk- AGCCTACGATCTGCGGTGCAAC  CYAGACATACATCAGATCAYATGTCTATTTATAAATTYAT | HepA-A1-LDR-DP-2c | GGGAAGGGGTCAYTTYTTGTGCACTTTTACHTTCA |
| Hep A-A1-LDR-UP-3 | Blk- ATGCGTAGCCCGTAACTTAGCC  GAGTACACATTTCCYATAACYYTGTCTTCAACCTCTAAT | HepA-A1-LDR-DP-3 | CCTCCTCATGGTYTRCCATCAACATTRAGGT |
| HepA-A2-LDR-UP-1 | Blk- CGGCGGAAGGTAGATCGTCCTCA  GATCAGATTRCCATGGTATTCTTAYTTRTAT | HepA- A2-DP-LDR1a | GCYGTKTCWGGRGCACTGGATGGT |
| HepA-A2-LDR-UP-2 | Blk-AGCTAGTCCGGGTACTGGGCAA  ATTCARATTGCAAAYTACAAYCAYTCWGAT | HepA- A2-DP-LDR2a | GAATAYTTGTCYTTTAGTTGYTAYTTGTCTGT |
| NoV-G1-A1-UP-LDR1a | Blk-ACCGCACTACAATTCCGGTGGG  TGGTRCCACACMMHCARAGGAAAGT | NoV-G1-A1-LDR-DP1a | WCAAYTGATCTCACTCYTGGGAGAGGC |
| NoV-G1-A1-UP-LDR1b | Blk-ACCGCACTACAATTCCGGTGGG  TGGTGCCACACAMMCAAAGRAAAGT | NoV-G1-A1-LDR-DP1b | CCAACTAATATCATTATTGGGTGAGGC |
| NoV-G1-A1-UP-LDR1c | Blk-ACCGCACTACAATTCCGGTGGG  CTCTGGTACCACACACCCAGAGGAAAGT | NoV-G1-A1-LDR-DP1c | CCAACTGATCTCACTCCTGGGAGAA |
|  |  | NoV-G1-A1-LDR-DP1d | ACAATTGATCTCACTCTTGGGAGAGGC |
| NoV-G1-A1-LDR-UP2a | Blk-TGACGGAATCCCTGTACGACGT  TGAAATYAAGACTGGWGGGYTGGAGAT | NoV-G1-A1-LDR-DP2a | GTSCCAGGRTGGCARGCCATGTT |
| NoV-G1-A1-LDR-UP2b | Blk-TGACGGAATCCCTGTACGACGT  GGAAATTAAAACAGGGGGCCTTGAAAT | Rev- NoV-G1-A1-DP2a | GTAYGTCCCAGGRTGGCAGGCCATGTT |
| NoV-G1-A2-UP-LDR1a | Blk-CCCAGAATGCGGTTCCAGTGCT  CAGCTGGTACCRGAGGYAAAYACAGCT | NoV-G1-A2-DP-LDR1a | GACCCCTTACCCATGGAACCYGT |
| NoV-G1-A2-UP-LDR1b | Blk-CCCAGAATGCGGTTCCAGTGCT  CARCTKGTGCCGGAGGTTAATAMWKCT | NoV-G1-A2-DP-LDR1b | GARCCTATWTCAATGGAGCCTGT |
| NoV-G1-A2-UP-LDR1c | Blk-CCCAGAATGCGGTTCCAGTGCT  CAGTTGGTACCGGAGGTTAATGCTTCT | NoV-G1-A2-DP-LDR1c | GACCCTCTTGCAATGGATCCTGT |
|  |  | NoV-G1-A2-DP-LDR1d | GAACCACTGCCWCTTGATCCKGT |
|  |  | NoV-G1-A2-DP-LDR1e | GACCCYATACCYATTGACCCTGT |
| NoV-G1-A2-UP-LDR2a | Blk-GCACTTTGGCGGGATTAGCAGA  CYGCTGGYCAAGTTAATATGATTGAYCCYT | NoV-G1-A2-DP-LDR2a | GGATYTTYAAYAAYTTTGTCCARGC |
| NoV-G1-A2-UP-LDR2b | Blk-GCACTTTGGCGGGATTAGCAGA  GCTGGRCAAGTTAAYATGATTGAYCCMT | NoV-G1-A2-DP-LDR2b | GGATAATSAATAATTWTGTDCAAGCC |
| NoV-G1-A2-UP-LDR2c | GCACTTTGGCGGGATTAGCAG  ACTGCTGGGCAAGTTAACCCTATTGACCCTT | NoV-G1-A2-DP-LDR2c | GGATAATYAATAAYTTTGTGCARGCTC |
| NoV-G1-A2-UP-LDR2d | Blk-GCACTTTGGCGGGATTAGCAG  ACWGCAGGCCARGTTAATTTGATTGATCCCT |  |  |
| NoV G2-A1-UP-LDR1a | Blk-TCAACCATGTCGAGCCTTCTGG  GCCAGGGCRACYGARGARGAYTTCTGT | NoV-G2-A1-DP-LDR1a | GAAGAGGARGAGGYCAAGATYCGRCA |
| NoV G2-A1-UP-LDR1b | Blk-TCAACCATGTCGAGCCTTCTGG  GCCAGAGCAACAGAAGAGAACTTTTGT |  |  |
| NoV G2-A1-UP-LDR1c | Blk-TCAACCATGTCGAGCCTTCTGG  GCCCGTGCAACAGAGGAGGATTTCTGT |  |  |
| NoV G2-A1-UP-LDR2 | Blk-CGGTTGACCAGTACCACGATTC  AAACAACGCAARGAGGARAGRGCYTCYCT | NoV-G2-A1-DP-LDR2a | CGGTTTRGTCACAGGYTCTGAAATYAGGA |
|  |  | NoV-G2-A1-DP-LDR2b | TGGCCTTGTCACHGGSTCAGAGATCAGAA |
|  |  | NoV-G2-A1-DP-LDR2c | GGGCCTAGTTACGGGCAGTGAGATAAGGA |
| NoV G2-A2-UP-LDR1 | Blk-TGGACCAGAGCACGGGTAGTTT  GTYCCAGAGGTCAAYAATGAGGTYAT | NoV G2-A2-DP-LDR1 | GGCTYTGGARCCCGTTGTTGRTGC |
| NoV G2-A2-UP-LDR2 | Blk- TGCTGATGACCGAGGCCAAGCT  ACCTGTRGCGGGCCAACAAAAYRTAAT | NoV G2-A2-DP-LDR2 | TGACCCCTGGATTAGAARYAAT |
| SaV-A1-UP-LDR1a | Blk- TTCCCGATACCGATTGCATCCG GTTGCYACTGGCWCMATCCAAT | SaV-A1-LDR-DP-1a | CAAATGTCCCTGAAGCGRTACGCAACTGT |
| SaV-A1-UP-LDR1b | Blk- TTCCCGATACCGATTGCATCCG GTTGCYACTGGTGCAAYCCAAT | SaV-A1-LDR-DP-1b | CSAAYGTCCCTGAGGCAATACGBAACTGT |
| SaV-A1-UP-LDR2a | Blk-AAGCCGCGTGAGTTCGTCCATG  CGYACTTTTGCTTGGAAYGACAGRAT | SaV-A1-LDR-DP-2a | GCCCRCGGGAACTTWYCTTGGATCTGT |
| SaV-A1-UP-LDR3a | Blk- CATTGGTCCGCACTTCAATCGC GGTAGTTTTGAAGCCCGCATCTCAAT | SaV-A1-LDR-DP-2b | GCCCACKGGAACTTTTCTKGGATCTWT |
| SaV-A1-UP-LDR3b | Blk- CATTGGTCCGCACTTCAATCGC GGYAGYTTTGAGGTCCGGYTATCGAT | SaV-A1-LDR-DP-3a | CTCTGGGTCTGGTGTSTWYGCTGGT |
| SaV-A1-UP-LDR3c | Blk- CATTGGTCCGCACTTCAATCGC GGCAGTTTTGAGGCCCGTCTATCGGT | SaV-A1-LDR-DP-3b | CTCTGGTTCTGGCGTGTTCGCTGGT |
| SaV-A1-UP-LDR3d | Blk- CATTGGTCCGCACTTCAATCGC GAGGTAGTTTTGAGTCCCGYGTTTCGAT | SaV-A1-LDR-DP-3c | TTCTGGGTCTGGCATGTTTGCTGGT |
| SaV-IV-A1-UP-LDR1 | Blk- TTCCCGATACCGATTGCATCCG CAWGCTCTYGACGTGCCAGGAACAACT | SaV-IV-A1-DP-LDR1 | GGYCCGACTTCGTCGGCAGTKGT |
| SaV-IV-A1-UP-LDR2 | Blk- AAGCCGCGTGAGTTCGTCCATG  CAGGCCCAACGYATGGARCTGGCT | SaV-IV-A1-DP-LDR2 | GTTGCAACCGGTGCCGTRTCATC |
| SaV-IV-A1-UP-LDR3 | Blk- CATTGGTCCGCACTTCAATCGC TGTCCCGGATGCAGTGCGTCART | SaV-IV-A1-DP-LDR3 | GCYTTGCACTCCTTCGCACATTTCCTT |
| SaV-V-A1-UP-LDR1a | Blk- TTCCCGATACCGATTGCATCCG TGCRGACGCACCCCTTGTACCTGTTAAT | SaV-V-A1-DP-LDR1a | CCAGAACAGCCCAATCTCCCTGCC |
| SaV-V-A1-UP-LDR2a | Blk- AAGCCGCGTGAGTTCGTCCATG  TGCCACAGGAGCAACAWCCTCCAAT | SaV-V-A1-DP-LDR2a | GTCCCTGASTGTGTGCGGAGYTGCT |
| SaV-V-A1-UP-LDR3a | Blk- CATTGGTCCGCACTTCAATCGC TGCTCTCCTTCGYACGATTCCTTGGAAC | SaV-V-A1-UP-LDR3a | ACTCGACAGCCCCAGGGATCTTTGC |

**Supplemental Table S1B**. LDR primers used for identification of enteric viruses.

Primer names indicate the virus detected as well as the amplicon of on which the LDR primer pairs were designed; and in some cases the genotype of the virus detected. For example, NoV G2-A2-UP-LDR1 is designed to detect NoV GGII and is designed on a nucleotide position on PCR amplicon 1. When more than one primer pair was designed for a particular virus, they were indicated with different numbers 1,2,3 etc. When multiple primers were designed at any LDR position an alphabetical suffix is included (for example, “a” or “b”). An amino blocking group (Blk) was used at the 5’end of the upstream primers and downstream primers were labeled with Cy-3 at the 3’ end. The first 22 nucleotides of the upstream primer (underlined) are the zipcodes complementary to the VeraCode™ micro- beads (Illumina, Inc. San Diego, CA).
